# Supplementary material for: Managing urban development could halve nitrogen pollution in China
Source: Nat Commun. 2024 Jan 9;15:401. doi: 10.1038/s41467-023-44685-y (PMC10776873; doi:10.1038/s41467-023-44685-y)
Supplement: Supplementary file 1 — Supplementary information [file 41467_2023_44685_MOESM1_ESM.pdf]

Supplementary information

Managing urban development could halve nitrogen pollution in China

Ouping Deng[1,2,#], Sitong Wang[1,3,#], Jiangyou Ran[2], Shuai Huang[2], Xiuming Zhang[1], Jiakun Duan[1], Lin Zhang[4], Yongqiu Xia[5], Stefan Reis[6], Jiayu Xu[4], Jianming Xu[1,7], Wim de Vries[8], Mark A. Sutton[9], Baojing Gu[1,3,10*]

[1]College of Environmental and Resource Sciences, Zhejiang University, Hangzhou 310058, China.
[2]College of Resources, Sichuan Agricultural University, Chengdu 611130, China.
[3]Policy Simulation Laboratory, Zhejiang University, Hangzhou 310058, China
[4]Department of Atmospheric and Oceanic Sciences, School of Physics, Peking University, Beijing 100871, China.
[5]Key Laboratory of Soil and Sustainable Agriculture, Changshu National Agr-Ecosystem Observation and Research Station, Institute of Soil Science, Chinese Academy of Sciences, Nanjing 210008, China.
[6]Unit for Environment and Sustainability at the German Aerospace Centre's Project Funding Agency, DLR Projekttraeger, Bonn 53227, Germany.
[7]Zhejiang Provincial Key Laboratory of Agricultural Resources and Environment, Zhejiang University, Hangzhou 310058, China
[8]Environmental Systems Analysis Group, Wageningen University & Research, Wageningen 91016700HB, The Netherlands.
[9]UK Centre for Ecology & Hydrology, Bush Estate, Penicuik, Midlothian EH26 0QB, UK.
[10]Ministry of Education Key Laboratory of Environment Remediation and Ecological Health, Zhejiang University, Hangzhou 310058, China

[#] These authors contributed equally

* bjgu@zju.edu.cn (B.G.)

This file inclues:

Supplementary Methods S1.1-S1.9

Supplementary Tables 1-11

Supplementary Figures 1-10

Contents

S1. Supplementary Methods 3
S1.1 Urban expansion modeling. 3
S1.2 Rural land reclamation modeling. 3
S1.3 The potential modeling of large-scale farming potential. 4
S1.4 Crop-livestock coupled production modeling 4
S1.5 Nitrogen budget modeling. 5
S1.6 High spatial resolution N loss inventory building. 6
S1.7 Air $PM_{2.5}$ estimation and validation 6
S1.8 Estimation of Nr output to sea and its imitation 6
S1.9 Geo-distribution of social benefit 8
Table S1 Land use change flow matrices from 2017 to 2050 (million ha). 9
Table S2 Pig unit conversion factor and the total livestock. 10
Table S3 Key parameters adjusting in the human relating subsystems in CHANS. 11
Table S4 Key parameter adjusting in agricultural relating subsystems in CHANS. 13
Table S5 Proxy variable and land use raster for high spatial resolution Nr loss inventory. 14
Table S6 Investment cost of sewage/garbage projects from Public-Private Partnership Service Platform of China. 15
Table S7 Investment cost of rural reclamation, cropland consolidation and livestock relocation .. 16
Table S8 GAINS mitigation unit cost (US$ kg N^{-1}) by provinces in 2017. 17
Table S9 N_r loss-related social benefit through urbanization in China 18
Table S10 Cost-benefit analysis of China's regional divisions. 18
Table S11 Constraint zone for livestock breeding and livestock volume through crop-livestock coupled 19
Fig. S1 | Mechanism of N loss reduction with urbanization. 20
Fig. S2 | Changes on population with urbanization. 20
Fig. S3 | Field size distribution through cropland consolidation and urbanization. 21
Fig. S4 | Livestock change through crop-livestock coupled. 21
Fig. S5 | Nitrogen emission in urban and rural waste treatment system 22
Fig. S6 | Geographic distribution of chemical N fertilizer and manure N input through agricultural optimization. 23
Fig. S7 | The Geographic distribution of N_2 emission with urbanization. 24
Fig. S8 | Geographic distribution changes of Nr emissions through three urbanization processes. 25
Fig. S9 | Methods for quantifying effects of urbanization on nitrogen pollution. 26
Fig. S10 | Comparison of observed and modeled monthly mean $PM_{2.5}$ concentrations.. 27
Supplementary References: 27

S1. Supplementary Methods

The High Resolution Remote Sensing Monitoring of Chinease Land Cover 2018 (HRRSM-CLC2018, 30 m), county-level population census and multi-source livestock database (FAOSTAT and APSCC2017) were combined to stimulate the urbanization processes in ArcGIS platform (version 10.6), including process of population migration, land use geo-distribution change, agricultural management change, and industrial upgrading. Then, the varying forms of Nr loss from the > 2,800 countries were calculated in 2017 and 2050 by the Coupled Human And Natural Systems (CHANS) model in China. Meanwhile, changes on air and water quality based on Nr loss reduction were simulated by the Weather Research and Forecasting model coupled with Chemistry (WRF-Chem) and the Water Nitrogen Retention and Removing model (WNRR), respectively. Finally, the policy feasible and cost-benefit of the urbanization pathway were analyzed. The framework of the method is shown in Fig. S9.

S1.1 Urban expansion modeling.

Since urban expansion occurs preferentially in the peri-urban grids, these grids have higher urban expansion priority (UEP_i)[1]. Focus Statistics Tool in ArcGIS was used to simulate UP_i with a value of 1 to the urban grid pixels and 0 to the other grids by the 100 by 100 grid rectangle.

$$UEP_i = \frac{U_i}{100\times100} \quad (1)$$

where U_i refers to the number of urban grids in the surrounding 100 by 100 grids centred on the ith grid. We ranked the UEP_i for all grids in descending order, and eliminate grids that are objectively unlikely to develop into cities (including current urban areas and water). If multiple girds have equal UEP_i , they are ranked according to GDP from largest to smallest, and by default regions with higher GDP are more likely to develop into cities. Based on the following linear plan, urban expansion grids are filtered out.

$$maxmize\ r$$

$$s.t. = \sum_{j=1}^{r} UP_j \le UP_{2050} \quad (2)$$

where j is the serial number ranked by UEP_i and GDP, and UP_j is the urban population in the jth grid, UP_{2050} is the national urban population in 2050 as projected by United Nations' World Urbanization.

S1.2 Rural land reclamation modeling.

Assuming that non-agricultural land would be reclaimed to cropland more preferentially in grids with a higher proportion of surrounding cropland, a proxy of reclamation priority (REP_i) was defined and calculated. Statistics Tool in ArcGIS was used to simulate RP_i with a value of 1 and 100 to the cropland and rural built-up land grids, respectively, by the 300 by 300 grid rectangle.

$$REP_i = \frac{C_i + R_i}{R_i} \quad (3)$$

where C_i and R_i denote the number of grids of cropland and rural land in 300 × 300 grids, respectively. We ranked all the rural grids in descending order of REP_i and eliminated the grids which were unsuitable for reclamation (slope > 25 degrees or elevation > 3000 m). Finally, based on the following linear programming, reclamation grids in 2050 were filtered out.

$$maxmize\ r$$

$$s.t. = \sum_{j=1}^{r} RP_j \le RP_{2050} \quad (4)$$

where j is the raster number ranked by RP_i, and RP_j is the rural population in the jth grid, RP_{2050} is the national rural population in 2050 as projected by United Nations' World Urbanization.

S1.3 The potential modeling of large-scale farming potential.

The high-resolution cropland raster data (30×30m) in 2050 was derived from the land reclamation modeling results. It is assumed that if there are no other features (e.g., rivers, roads, etc.) spatially separating the cropland grids within the county, they can be connected into larger croplands. We first split the raster data using county boundaries, then we used a raster data set to obtain polygon features of every cropland plot through Analysis Toolbox on ArcGIS version 10.6. Then, the plots were divided into five categories (<0.64, 0.64-2.56, 2.56-16, 16-100, and >100 ha) according to the size of the field plots, and we analyzed the total number of plots and total area of each category. In particular, a grid of points with an interval of 1 km was produced. At each point, the k-nearest neighbour method was applied to the given point, and we set k=5 as suggested to obtain the value of the unknown point. The data analysis was done in R environment. The following R packages were used: raster (https://CRAN.R-project.org/package=raster); RANN (https://CRAN.R-project.org/package=RANN); and sp (https://CRAN.R- project.org/package=sp). Finally, the plot number and total area of five categories in the sub-counties were extracted, and the average field size in the sub-counties was estimated. More details can be found in Duan., 2021[2].

S1.4 Crop-livestock coupled production modeling

The current distribution of livestock manure and manure carrying capacity of large scale cropland was estimated in the grid scale. Based on FAOSTAT2017 and NBSC2017 database, numbers and manure N of 17 livestock and poultry species were collated (Table S2). Around 2.3 billion livestock were allocated in equal proportions according to APSCC2017 (5 livestock species, equaling 1.2 billion pig units in total). APSCC2017 covers more than 20,000 farm households and belongs to the Chinese Ministry of Agriculture and Rural Affairs[3]. Coefficients of Nr cycling in livestock systems, i.g. emission factor of NH_3 volatilization, ratio of manure return to the field, etc., also refer to the result of the APSCC2017[3]. The maximum livestock carrying capacity per hectare of cropland is around 30 pig units, equivalent to 117 kg N, referring to the Ministry of Agriculture's recommendation for manure carrying capacity in cropland[4]. If the manure N produced by livestock in the county is larger than the manure N carrying capacity of cropland, the livestock needs to be moved outside the county or province. Here, *LRPi* was used to quantify this process. *LRPi* is less than 0 means that the livestock carrying capacity of the grid is overload and the livestock need to be removed. While, it is greater than 0 means that the grid can still carry more livestock and can be moved into the livestock. Then, the provincial livestock relocation capacity ($PLRP_r$) and relocation amount (PRA_r) were calculated.

If *PLRP*>*PRA*, which represent the provincial livestock manure N can be carried by cropland, livestock relocation would be conducted inner province.

$$PLRP_r = \sum_{i=1}^{n} LRP_i, LRP_i \geq 0 \tag{5}$$

$$PRA_r = \sum_{i=1}^{n} LRP_i, LRP_i \leq 0 \tag{6}$$

S1.5 Nitrogen budget modeling.

The Couple Human and Natural System (CHANS) model was applied to calculated the nitrogen budget among 14 ecosystems with urbanization. Nitrogen mass balance is used to calculate and quantify N inputs and outputs, and then to determine if the system is an N source (input < output) or a sink (output < input) . The N balance calculations of the whole system, and for each subsystem follow the basic principle:

$$\sum_{h=1}^{m} IN_{\mathrm{h}} = \sum_{g=1}^{n} OUT_g + \sum_{k=1}^{p} ACC_{\mathrm{k}} \tag{7}$$

where IN_h and OUT_g represent N inputs and outputs, respectively, and ACC_k represents the different N accumulation terms. Most N input cycles into different subsystems, for example NO_x emission from fossil fuel combustion further deposits onto three major landscapes, natural land (i.e., forest, grassland), water bodies and cropland. The nitrogen cycling processes within and between different subsystems are described in detail in past studies[5, 6]. In this study, to make the human system more responsive to urbanization, the original human system was divided into urban and rural subsystems (Fig. S3). At the same time, nitrogen cycling processes were constructed for the urban and rural systems (Equations 8-11), and activity data and parameters were collected (see Table S11 for details).

$$UIN = \sum_{a=1}^{m} UFood_a + \sum_{b=1}^{m} UIndu_b + UFue \tag{8}$$

$$UOUT = \sum_{c=1}^{m} UWTP_c + \sum_{d=1}^{m} UGT_d \tag{9}$$

$$RIN = \sum_{a=1}^{m} RFood_a + \sum_{b=1}^{m} RIndu_b + RFuel + RStr \tag{10}$$

$$ROUT = \sum_{c=1}^{m} RWTP_c + \sum_{d=1}^{m} RGT_d + \sum_{e=1}^{m} NOx_e + OUT_{com} + OUT_{runoff} \tag{11}$$

Where *UIN* and *RIN* represent the input of nitrogen into urban and rural subsystems, respectively. *UOUT* and *ROUT* represent the output of nitrogen from urban and rural subsystems, respectively. $UFood_a$ and $RFood_a$ refer to the consumption of human food in urban and rural, including crop, livestock product (meat and milk) and

aquaculture product (fish and others), respectively. $UIndu_b$ and $RIndu_b$ represent the consumption of industrial product in urban and rural, respectively. *UFue* and *RFuel* represent the the consumption of fossil fuels in urban and rural, respectively. RStr refer to the straw input in rural. $UWTP_c$ and $RWTP_c$ refer to the domestic wastewater collected from urban and rural and treated by wastewater treatment plant, respectively. UGT_d and RGT_d are domestic garbage collected by urban and rural and disposed of by sound waste treatment methods (i.e., landfill, incineration and other), respectively. NOx_e refers to NO_x emission from fuel and straw consumption. OUT_{com} refers to the nitrogen content of compost from rural domestic garbage, while OUT_{runoff} refers to the nitrogen that leaches into waters from direct wastewater discharge and discarded garbage.

S1.6 High spatial resolution N loss inventory building.

Annual N emissions, including NH_3, NO_x, N_2O, and NO_3^-, derived from each subsystem of the CHANS model were assigned to counties by proxy parameters. Among them, the results of Wang et al. 2021[7, 8] were used for the inventory of ammonia emissions from cropland because of the large emissions and complex influencing factors. The geo-distribution of NH_3 emission from livestock was from APSCC2017. Then, emissions from each subsystem were assigned to the corresponding land-use raster on a county basis, plotted as a set of high-resolution emission inventory. For example, ammonia emission from chemical and organic fertilizers is assigned to agricultural land, and ammonia emission from industry and urban is assigned to urban. The proxy parameters and allocation raster for the spatial inventory of Nr emissions are shown in Supplementary Table S5.

S1.7 Air $PM_{2.5}$ estimation and validation

We used the WRF-Chem model to assess surface concentrations of $PM_{2.5}$ in 2017 and 2050. The model covers China and its surrounding area with a horizontal resolution of 27 km and 37 vertical layers extending from a 40 m thick surface layer to 50 mbar (mb)[9]. We drive the model with initial and boundary conditions from the 2017 atmospheric chemistry. Meteorological initial and lateral boundary conditions were provided by the National Centers for Environmental Prediction Final Global Run Analysis data at a resolution of 1°× 1°. Chemical initial and boundary conditions are archived from the Community Atmospheric Model with Chemistry(CAM-Chem). We simulated January, April, July, and October for 2017 and 2050, and assumed annual average concentrations as the average of these four months. We restarted WRF-Chem every 48 hours to prevent the drift effect of the simulated meteorological field. We assessed simulated 2017 surface $PM_{2.5}$ concentrations against ground-based observations from the Ministry of Ecology and Environment (MEE). Monitoring sites with monthly data missing rate less than 5% of monitoring site data were selected for model validation. Fig. S10 shows that the model well simulates the monthly variation and spatial pattern of surface $PM_{2.5}$ concentrations in mainland China.

S1.8 Estimation of Nr output to sea and its imitation

With the inventory of Nr loss to surface water as the primary data source, we estimated national Nr output to sea based on a water network-based framework (WNF)[10], which incorporates topology structure, hydrological and biogeochemical processes. It assumes that the Nr entering the surface water body, through a combination of linear water bodies (LW) and isolated areal water bodies (AW), eventually enters the ocean. Loading of Nr in *i*th grid to sea ($NtoS_i$) were estimated as follow:

$$NtoS_i = S_i \times \prod_{j=1}^{n} R_{lj} \times R_a \tag{12}$$

where S_i refers Nr output to inland water in *i*th grid calculating by CHANS. R_l and R_a were the released factor of Nr during linear water bodies and areal water bodies, respectively. *j* refers the different Strahler Order for different linear water bodies. We calculate the released factor of Nr during water transport (R_l and R_a) by letting each unit of travel time (t) have a dynamic removal rate (k) of the Nr it receives[11].

$$R = \exp(-kt) \tag{13}$$

The calculation of k includes the importance of water size, location, and water type as described in Xia and Yan (2020)[12] and Alexander et al (2000)[13]. And, t represent the travel time of Nr from a given grid *i to sea.* The travel times of linear water bodies (t_l) are assumed to increase as the length l_i the hydraulic path increases and as the square root of the average slope of the hydraulic path decreases (Ferro and Porto,2000). The travel times of areal water bodies (t_a) depends on the total area of the water (A_a) through which the Nr pass[11].

$$t_l = \frac{L}{v} = \frac{1}{n}\sum_{j=1}^{n} \frac{l_{i,j}}{R_{i,j}^{2/3}\sqrt{s_{i,j}}} \tag{14}$$

$$t_a = \beta_1 (A_a)^{\beta_2} \tag{15}$$

in which $l_{i,j}$ $R_{i,j}$ and $s_{i,j}$ are the length, hydraulic radius, and average slope of *i*th source during *j*th type of hydraulic path, respectively. β1 = 1.50, β2= 0.23 are the coefficients derived from a meta-analysis of different areal waters across the wold[11].

This framework is simple and computationally efficient, and can describe the influence of the topological structure of surface water on nutrient transport and retention. It can be applied to any available water structure, land use, and DEM map, without limitation on the size of the water body. Although this framework has been used and validated in the Yangtze River Basin[10], there are still some limitations in using it for nitrogen input estimation at the scale of China. For example, this framework does not consider the influence of water flow velocity on nutrient retention. In addition, it does not take into account the contribution of groundwater runoff to the ocean, and cannot evaluate the lagged benefits resulting from changes in nitrogen

input to rivers. Therefore, it may underestimate the nitrogen output to the ocean in 2050.

S1.9 Geo-distribution of social benefit

The total health benefits of the China are allocated to counties based on changes in air and water pollution (Fig. 5). The health benefit would be affected by the Value of Statistical Life (VSL) and county population density. For example, higher VSL and population density increase the health benefit10.5s per reduction of unit Nr loss, such as Beijing-Tianjin region. Therefore, the counties' health benefits (HB_t) are corrected using VSL and population density in 2050 with the following equation:

$$HB_t = \sum_q (HB_q \times \frac{\Delta PR_{m,t}}{\Delta PR_m} \times HE_q) \quad (16)$$

$$HE_q = \alpha \times \beta \times HX_q \quad (17)$$

where t represents >2800 counties. q represents the forms of Nr, i.e. NH_3, NO_x and NO_3^-. HB_q is the total health benefit on the reduction of the qth forms of Nr. m represents air or water pollution, denoted by $PM_{2.5}$ and river N loading, respectively. ΔPR_m is the total reduction of $PM_{2.5}$ or river N loading. HX_q represent the correction coefficients of health benefit. α, β represent the correction coefficient of VSL and population density, respectively, and are equal to the county value divided by the national average value.

As health benefits, ecosystem and climate benefits also are allocated at the county scale based on changes of Nr loss reduction. Considering that the county distribution of ecosystem benefit (EB_t) is influenced by the local economic status and environmental pollution status, while climate benefit (CB_t) is only influenced by the local economic status. GDP per capita and Nr loss in 2017 in county scale are used to make corrections for EB_t . The specific calculation formula are as follows:

$$EB_t = \sum_q (EB_q \times \frac{\Delta PR_{q,t}}{\Delta NR_q} \times EE_q) \quad (18)$$

$$EE_q = \eta^{\frac{1}{2}} \times \delta^{\frac{1}{3}} \times EX_q \quad (19)$$

$$CB_t = \sum_q (CB_q \times \frac{\Delta PR_{q,t}}{\Delta NR_q} \times CE_q) \quad (20)$$

$$CE_q = \eta \times CX_q \quad (21)$$

where ΔNR_q is the reduction of qth Nr through urbanization in China. EXq and CXq are the correction coefficients of ecosystem benefit and climate benefit, respectively. η, δ are the correction coefficient of GDP per captal and Nr emission in 2017, respectively.

Table S1 Land use change flow matrices from 2017 to 2050 (million ha).

2017-2050	Grassland	Urban	Cropland	Ocean	Forest	Rural	Water	Other	Areas in 2017
Grassland	219.2	0.1							219.3
Urban		5.5							5.5
Cropland		1.3	133.6						134.9
Ocean				0.0					0.0
Forest		0.4			252.5				252.8
Rural		0.3	6.9			6.2			13.4
Water							35.5		35.5
Other		0.2						58.3	58.5
Areas in 2050	219.2	7.8	140.5	0.0	252.5	6.2	35.5	58.3	719.9

Table S2 Pig unit conversion factor and the total livestock.

No.	Species	Pig unit conversion factor	FAOSTAT and NBSC		APSCC2017	
			(million head)	(million pig unit)	(million head)	(million pig unit)
1	Chickens, broilers	0.02	10298	165	8925	143
2	Chickens, layers	0.07	737	49	1906	126
3	Duck, brolilers	0.01	2404	22		
4	Duck,laying	0.08	737	62		
5	Groose	0.09	651	59		
6	Rrabbit	0.10	320	33		
7	Swine, market	1.00	712	712	674	674
8	Swine, breeding	0.57	447	254		
9	Dairy cattle	10.00	62	620	8	84
10	Other cattle	5.00	39	196	37	184
11	Buffalo	10.09	4	44		
12	Goat/Sheep	0.33	302	100	302	100
14	Horse	9.09	3	31		
15	Ass	4.95	3	13		
16	Mule	4.95	1	4		
17	Camel	8.27	0	3		
SUM			16720	2365	11853	1311

Note: This table shows the total nation livestock from three database, based on pig unit conversions. Pig unit conversion factors of 6 species of livestock collected in APSCC2017 were used in this study. Conversion factors for other animals were defined as the ratio of livestock excretion factor to swine excretion factor, calculated from FAOSTAT and NBSC.

Table S3 Key parameters adjusting in the human relating subsystems in CHANS.

Subsystem	Parameters	2017	2050	Method and parameters in 2050
Urban	Population (million person)	8.1	10.9	Population in 2050 is projected by The United Nations' World Urbanization Prospects
	Urban excretion (Tg N)	5.0	6.5	The volume of garbage production changes in proportion to the population between 2017 and 2050.
	# Disposal	4.1	6.3	The urban excretion disposal rates in 2050 are constant with the 14th Five-Year plan.
	# Leakage	0.4	0.0	
	Urban garbage production (Tg)	21521	28696	
	#Landfill	12038	10044	In 2050, the amount of urban garbage will be determined by multiplying the per capita urban garbage production rate by the urban population. Historical data shows that the per capita urban garbage production rate is 0.72 kg per person. Additionally, the latest (2021) National Statistical Yearbook of Urban Construction indicates the rate of environmentally sound disposal.
	#Incineration	8463	18653	
	#Other treatment	533	-	
	#Composting	-	-	
	#Discard	487	-	
Rural	Population (million person)	5.80	2.70	Population in 2050 is projected by The United Nations' World Urbanization Prospects
	Rural excretion (Tg N)	3.1	1.4	The volume of rural excretion changes in proportion to the population between 2017 and 2050.
	#Disposal	0.3	0.3	The rural excretion disposal rates in 2050 are referenced in the 14th Five-Year plan. The rates of untreated and returned to cropland wastewater in 2050 were determined based on their respective shares in the remaining
	#Return to cropland	1.0	0.4	
	#Untreated	1.0	0.4	

	#Leakage	0.3	0.1	parts in 2017.
	Rural garbage production (Tg)	19994	9449	
	#Landfill	3514	767	The calculation of waste generation in rural areas follows the same method as in urban areas, with the per capita waste generation rate in rural areas being 0.8 kg per person, according to data from the National Ministry of Health survey. Meanwhile, the rate of environmentally sound disposal in 2050 is consistent to 2017.
	#Incineration	2470	2653	
	#Other treatment	78	119	
	#Composting	2178	1029	
	#Discard	7319	2823	
	#Kitchen garbage feed	4357	2059	
Wastewater	Domestic sewage discharge (billion m3)	59	86	The quantity of domestic wastewater has increased by 1.3 times compared to that in 2017, primarily due to changes within the Urban and Rural subsystems domain.
Urban greenland	Greenland area (million ha)	2.9	4.3	Areas increase by 48% in proportion to urban expansion rate.

Note: Data for 2017 for both urban and rural areas are from the 2018 National Year of Urban Construction Statistics

Table S4 Key parameter adjusting in agricultural relating subsystems in CHANS.

Subsystem	Parameters	2017	2050	Method and reference for parameters in 2050
Cropland	N fertilizer (Tg)	28.9	10.1	Nitrogen fertilizer input changes through three steps. First, it would increase by 4% in corporation with cropland areas. Second, it would decrease by 38% due to large-scale farming. Finally, it would decrease by 46% due to more manure returned to the cropland.
	NH_3 emission factor	11%	9%	NH3 emission factor decreased by 17% due to large-scale farming8.
	Straw N (Tg N)	5.9	6.1	
	#Recycle to cropland	3.5	3.5	
	#Livestock feed	1.1	2.2	By 2030, 57% of cropland is projected to be converted into high-standard farmland, with a 100% straw recycling rate. This means 57% of straw will be recycled and used in cropland. Additionally, as a result of crop-livestock coupling, any remaining straw will be used as feed for livestock.
	#Domestic straw fuel	0.9	0.0	
	#Industrial materials	0.4	0.4	
	#Burning in field	0.0	0.0	
Livestock	Manure applied to cropland	5.5	10.1	Whole manure would return to cropland after crop-live stock coupled

Table S5 Proxy variable and land use raster for high spatial resolution Nr loss inventory.

Subsystem in CHANS	Proxy Variable	Land use raster
Cropland	Wang et al., 2021[7, 8]	Cropland
Livestock	Zhu et al., 2022[3]	Cropland, Rural
Grassland	Grassland area	Grassland
Aquaculture	Water area	Water
Urban greenland	Urban area	Urban
Human-urban	Population of urban	Urban
Human-rural	Population of rural	Rural
Industry	Industrial output	Urban
Wastewater	Population	Urban, Rural
Solid waste	Population	Urban, Rural
Forest	Forest area	Forest

Note: The portion of livestock manure returned to cropland is allocated to cropland, while the rest of manure is allocated to rural.

Table S6 Investment cost of sewage/garbage projects from Public-Private Partnership Service Platform of China.

Region code	Investment projects for sewage treatment			Investment projects for garbage landfill			Investment projects for garbage incineration		
	No. of projects	Mean (US\$ m^{-3})	Range (US\$ m^{-3})	No. of projects	Mean (million US\$ t^{-1})	Range (million US\$ t^{-1})	No. of projects	Mean (million US\$ t^{-1})	Range (million US\$ t^{-1})
PIR	26	1875	403 - 6977	1	0.06	0.06-0.06	5	0.09	0.06-0.11
DOR	93	1955	262 - 26833	15	0.09	0.02-0.34	25	0.14	0.02-1.77
LOR	29	1222	522 - 4186	12	0.10	0.03-0.16	6	0.10	0.00-0.23

Taking into account both population migration and economic development level, China was divided into three regions for investment assessment: a population inflow region (PIR), a developed outflow region (DOR) and a less developed outflow region (LOR), as detailed in Table S10.

Table S7 Investment cost of rural reclamation, cropland consolidation and livestock relocation

Region code	Rural reclamation			Cropland consolidation		
	Number of projects	Rang (US$ ha^{-1})	Mean (US$ ha^{-1})	Number of projects	Rang (US$ ha^{-1})	Mean (US$ ha^{-1})
PIR	7	36225-979054	337731	46	2275-12516	5196
DOR	20	2-323023	54481	116	1725-11796	4528
LOR	2	13-22007	21654	39	1243-6604	3546
Region code	Livestock facilities dismantling			Subsidy of livestock		
	Number of projects	Rang (US$ ha^{-1})	Mean (US$ ha^{-1})	Number of projects	Rang (US$ pig^{-1})	Mean (US$ pig^{-1})
PIR	5	6.4-69.7	29.2	10	9.7-51.5	35.4
DOR	7	3.2-56.4	24.9	7	6.4-32.2	16.8
LOR	NA	-	26.7	NA	-	27.8

Note: Cost data of homestead reclamation and cropland consolidation were collected from more than 200 implemented projects in China Land Consolidation and Rehabilitation (CLCR, http://www.lcrc.org.cn). Subsidy of livestock relocation were collected from 35 local compensation programs by local governments. Taking into account both population migration and economic development level, China was divided into three regions for investment assessment: a population inflow region (PIR), a developed outflow region (DOR) and a less developed outflow region (LOR), as detailed in Table S10.

Table S8 GAINS mitigation unit cost (US$ kg N^{-1}) by provinces in 2017.

Province	Abbreviation	Mitigation unit cost of industrial upgrading		Province	Abbreviation	Mitigation unit cost of industrial upgrading	
		NH_3	NO_x			NH_3	NO_x
Beijing	BJ	2.30	25.56	Hubei	HB	1.65	10.30
Tianjin	TJ	2.11	33.10	Hunan	HN	2.03	11.43
Hebei	HB	1.53	20.48	Guangdong	GD	1.13	4.27
Shanxi	SX	2.76	15.01	Guangxi	GX	2.08	8.77
Inner Mongolia	NMG	2.18	17.85	Hainan	HN	1.99	2.06
Liaoning	LN	2.33	22.91	Chongqing	CQ	2.38	5.77
Jilin	JL	1.72	16.47	Sichuan	SC	2.49	6.09
Heilongjiang	HLJ	2.27	16.80	Guizhou	GZ	2.41	11.04
Shanghai	SH	2.40	4.99	Yunnan	YN	2.23	5.83
Jiangsu	JS	1.06	4.92	Tibet	XZ	2.05	0.46
Zhejiang	ZJ	1.32	5.62	Shannxi	SX	4.11	15.12
Anhui	AH	1.61	12.04	Gansu	GS	3.25	23.55
Fujian	FJ	2.18	5.94	Qinghai	QH	4.01	8.19
Jiangxi	JX	2.39	11.89	Ningxia	NX	3.96	18.07
Shandong	SD	1.95	9.99	Xinjiang	XJ	3.21	13.26
Henan	HN	1.37	13.76				

Note: original cost data are derived from Zhu et al. (2022)[3] and Zhang et al. (2020)[14]

Table S9 N_r loss-related social benefit through urbanization in China

	Unit benefit (US$ kg N^{-1})			Total benefit (US$ billion)		
	Human health	Ecosystem	Climate	Human health	Ecosystem	Climate
**NH_3**	4.2	6.3	-1.5	-26.3	-39.4	9.4
**NO_x**	18.7	8.1	-3.6	-33.1	-14.3	6.4
**N_2O**	1.1	1.1	11.9	-0.6	-0.6	-6.3
**NO_3^-**	2.0	13.2	0.0	-15.0	-101.4	0.0
**SUM**				-74.9	-155.7	9.5

Note: original unit cost data are derived from Zhu et al. (2022)[3]

Table S10 Cost-benefit analysis of China's regional divisions.

Region code	Explanation	Contains province
PIR	Inflow region	Beijing, Tianjin, Shanghai, Jiangsu, Zhejiang, Fujian, Guangdong, Chongqing
DOR	Developed outflow region	Hebei, Shanxi, liaoning, Anhui, Jiangxi, Shandong, Henan, Hubei, Hunan, Guangxi, Hainan, Sichuan, Guizhou, Shannxi
LOR	Less developed outflow region	Inner Mongolia, Jilin, Heilongjiang, Yunnan, Tibet, Gansu, Qinghai, Ningxia, Xinjiang

Note: The region division is based on population change during urbanization and GDP per capta of each provinces.

Table S11 Constraint zone for livestock breeding and livestock volume through crop-livestock coupled

Province	Abbreviation	Zones for livestock production	Livestock (million pig unit)		Province	Abbreviation	Zones for livestock production	Livestock (million pig unit)	
			2017	After coupled				2017	After coupled
Beijing	BJ	Constraint zone	8	8	Hubei	HB	Constraint zone	142	142
Tianjin	TJ	Constraint zone	17	15	Hunan	HN	Constraint zone	173	117
Hebei	HB		184	186	Guangdong	GD	Constraint zone	126	76
Shanxi	SX		81	92	Guangxi	GX		128	96
Inner Mongolia	NMG		96	168	Hainan	HN		20	17
Liaoning	LN		132	144	Chongqing	CQ		54	60
Jilin	JL		67	91	Sichuan	SC		179	198
Heilongjiang	HLJ		89	89	Guizhou	GZ		73	67
Shanghai	SH	Constraint zone	3	3	Yunnan	YN		185	108
Jiangsu	JS	Constraint zone	108	108	Tibet	XZ		14	4
Zhejiang	ZJ	Constraint zone	32	32	Shannxi	SX		60	104
Anhui	AH	Constraint zone	109	109	Gansu	GS		60	66
Fujian	FJ	Constraint zone	57	32	Qinghai	QH		17	12
Jiangxi	JX	Constraint zone	90	85	Ningxia	NX		31	16
Shandong	SD		227	235	Xinjiang	XJ		82	121
Henan	HN		202	247					

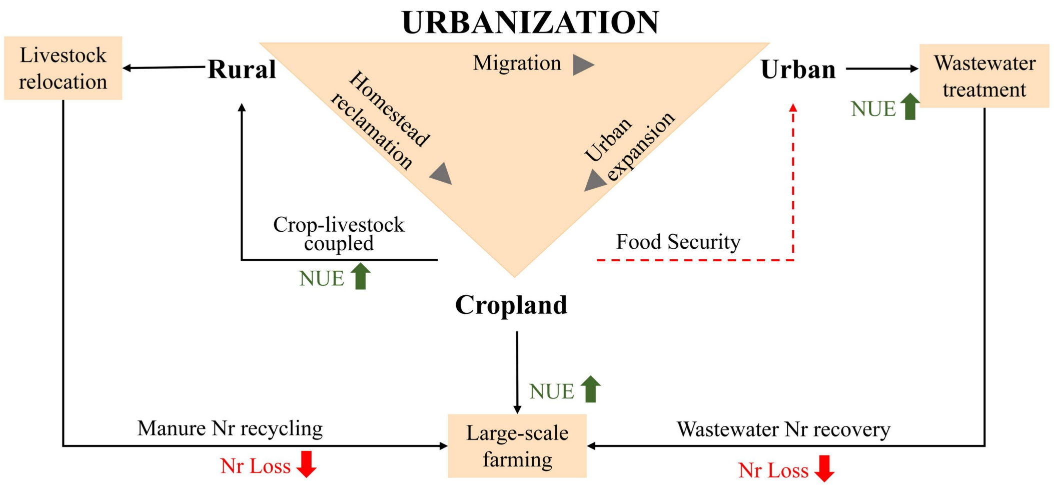

Fig. S1 | Mechanism of N loss reduction with urbanization.
Rural-urban migration benefits the management of domestic sewage and waste due to centralized waste management and pollution control measures. Meanwhile, quantity and scale of croplands and the coupling of crop and livestock production would be increased according to rural reclamation and rural population reduction. These processes are beneficial for improving N use efficiency (NUE) and reducing N losses.

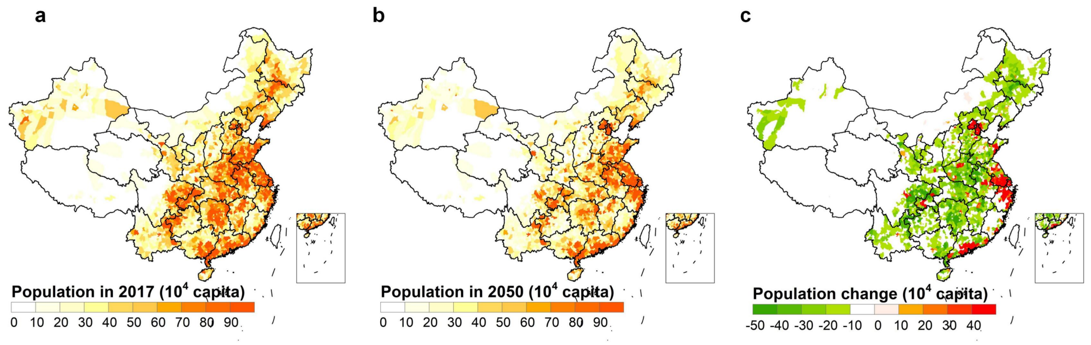

Fig. S2 | Changes on population with urbanization.
a-c, Geo-distribution of population in 2017, 2050 and the difference. The base map of China is derived from the Database of Global Administrative Areas (GADM, https://gadm.org/).

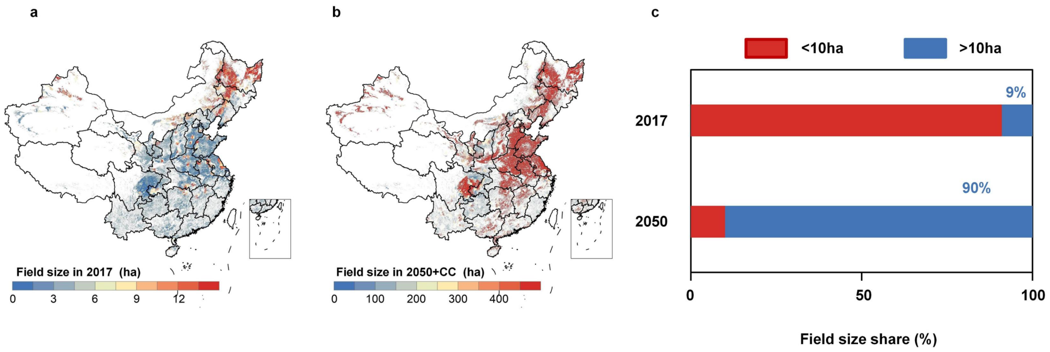

Fig. S3 | Field size distribution through cropland consolidation and urbanization. **a**-**c**, The geographic distribution of cropland field size in 2017 (**a**), and integration of consolidation and urbanization in 2050 (**b**). **c**, Field size share with cropland consolidation and urbanization. Data of current field size in 2017 and potential field size after cropland consolidation were derived from Lesiv et al., 2018[15] and Duan et al., 2021[2], respectively.

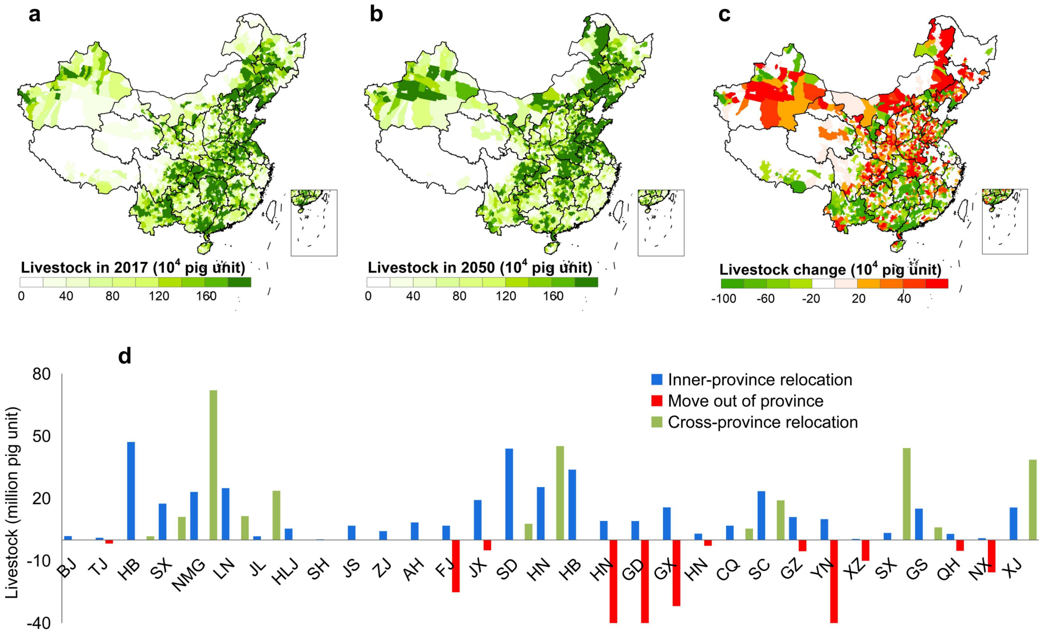

Fig. S4 | Livestock change through crop-livestock coupled.

Followed by the distribution of large-scale farms in 2050, livestock would be relocated with constrains that livestock manure production does not exceed the carrying capacity of crop requirement on county scale. **a-c**, Geographic distribution of livestock in 2017 (**a**), 2050 (**b**) and difference (**c**). **d**, The number of livestock moving into the province, out of the province, and relocation within province in 31 provinces. The full names of 31 provinces are shown in table S6.

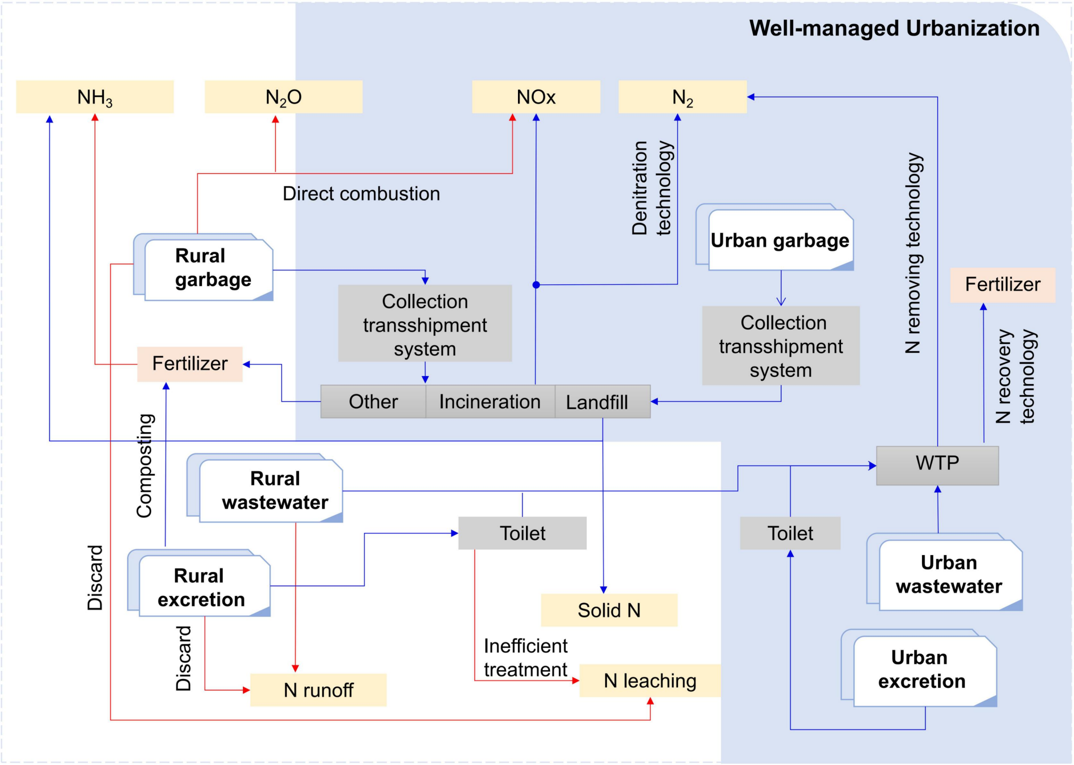

Fig. S5 | Nitrogen emission in urban and rural waste treatment system
The diagram includes traditional and well-managed urban and rural waste treatment systems. The red arrows represent traditional treatment path; the blue arrows represent harmless treatment methods. The yellow box represents the final discharge form of nitrogen; the gray box represents ancillary facilities in the harmless treatment process, including toilets, waste transfer systems, wastewater treatment plants (WTP), landfills, and incineration plants; the white boxes represent types of waste from urban or rural areas.

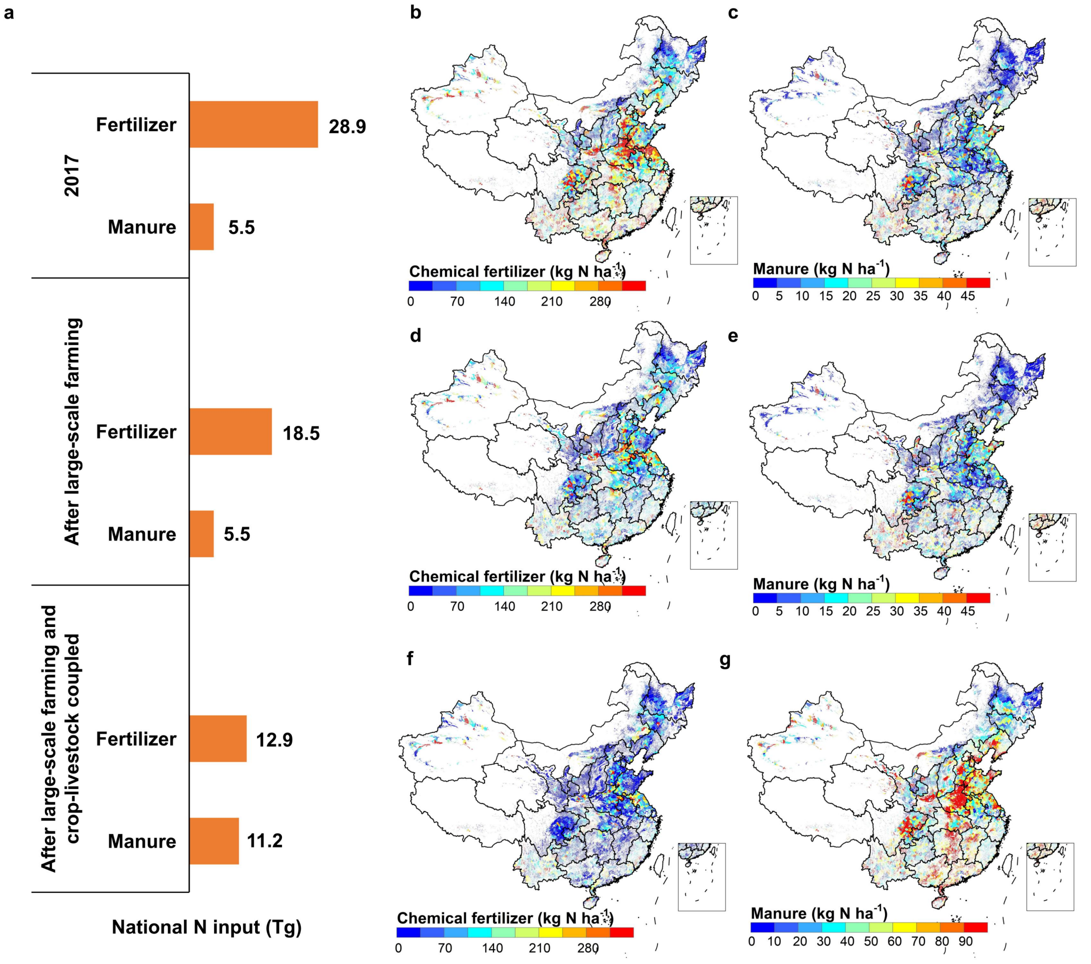

Fig. S6 | Geographic distribution of chemical N fertilizer and manure N input through agricultural optimization.

Agricultural optimization includes large-scale farming and crop-livestock coupled production. **a**, National scale input quantities of chemical and manure N fertilizer. **b-c**, Geographic distribution of chemical and manure N fertilizer input in 2017. **d-e**, Geographic distribution of chemical and manure N fertilizer input after large scale farming. **f-g**, Geographic distribution of chemical and manure N fertilizer input after large scale farming and crop-livestock coupled.

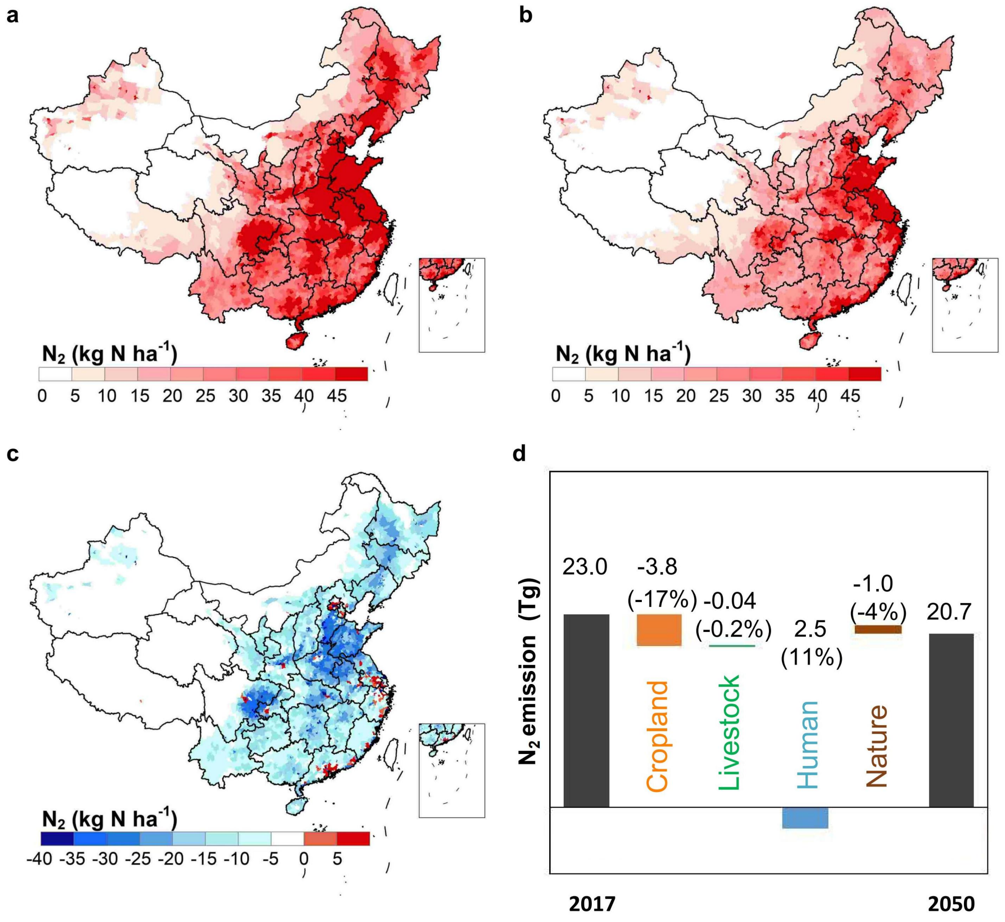

Fig. S7 | The Geographic distribution of N_2 emission with urbanization.
a-c, Spatial distribution of N_2 emissions in 2017 (**a**) and in 2050 (**b**), and the difference (**c**). d, National N_2 emissions in 2017 and 2050, and the sources of the difference.

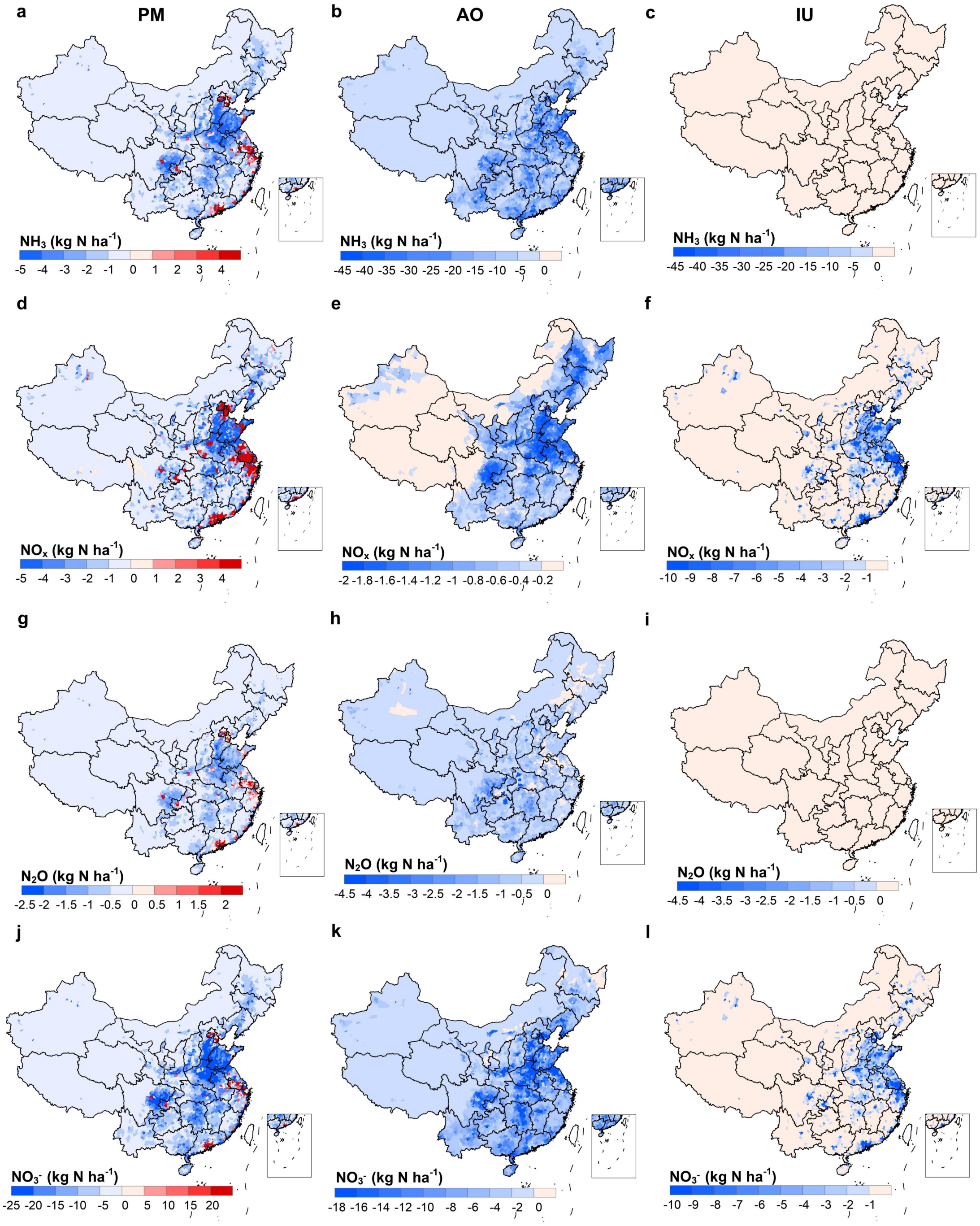

Fig. S8 | Geographic distribution changes of Nr emissions through three urbanization processes.
a-c, Geographic distribution changes of NH_3 through population migration(PM), agricultural optimization(AO) and industrial upgrading(IU), respectively. **d-f**, Geographic distribution changes of NO_x through PM, AO and IU, respectively. **g-i**, Geographic distribution changes of N_2O through PM, AO and IU, respectively. **j-l**, Geographic distribution changes of NO_3^- through PM, AO and IU, respectively. Since industrial upgrading primarily focuses on reducing NO_x emissions and has little impact on NH_3 and N_2O emissions, there is minimal change in **c** and **i**.

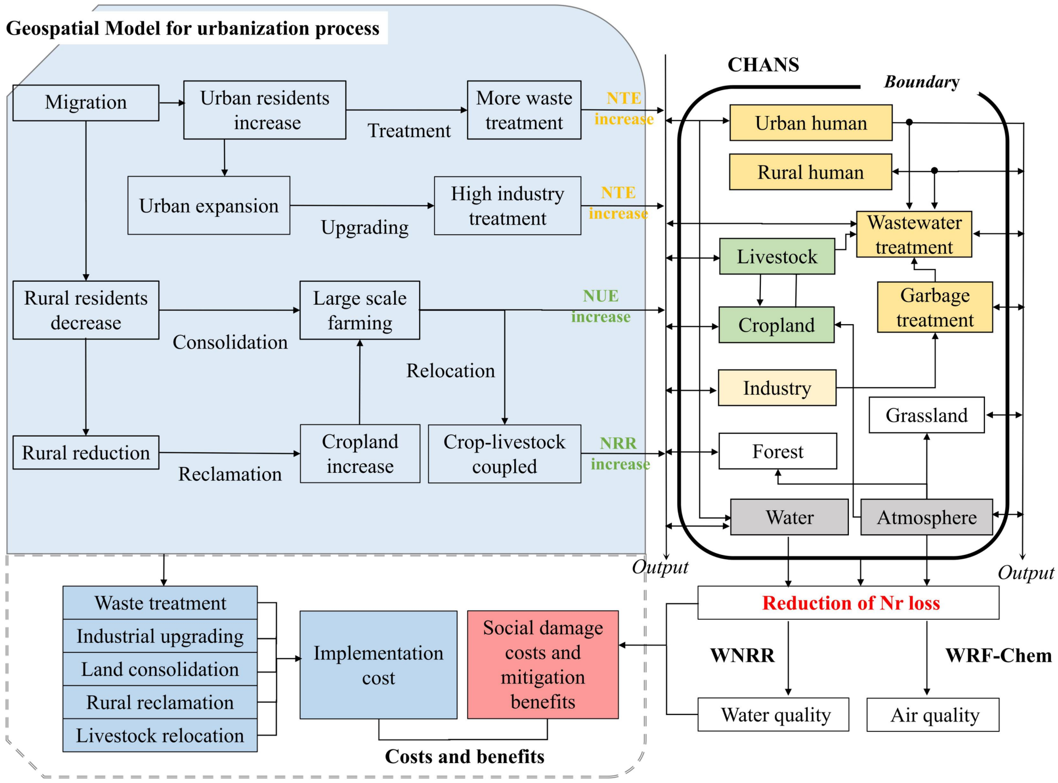

Fig. S9 | Methods for quantifying effects of urbanization on nitrogen pollution. Graphical Model for urbanization process represents the whole simulation process, including migration, urban expansion, industrial upgrading, cropland consolidation, livestock relocation, and rural reclamation. It changes the whole nitrogen cycling on national scale. The urbanization process covering the period from 2017 to 2050 would eventually reduce Nr losses, which enhance air and water quality. The red boxes represent the estimation of costs incurred through urbanization. Abbreviations: NTE, Nitrogen Treatment Efficiencies;NUE, Nitrogen Use Efficiency; NRR, Nitrogen Recycle Ratio; WNRR, Water Nitrogen Retention and Removing model; WRF-Chem, Weather Research and Forecasting model coupled with Chemistry.

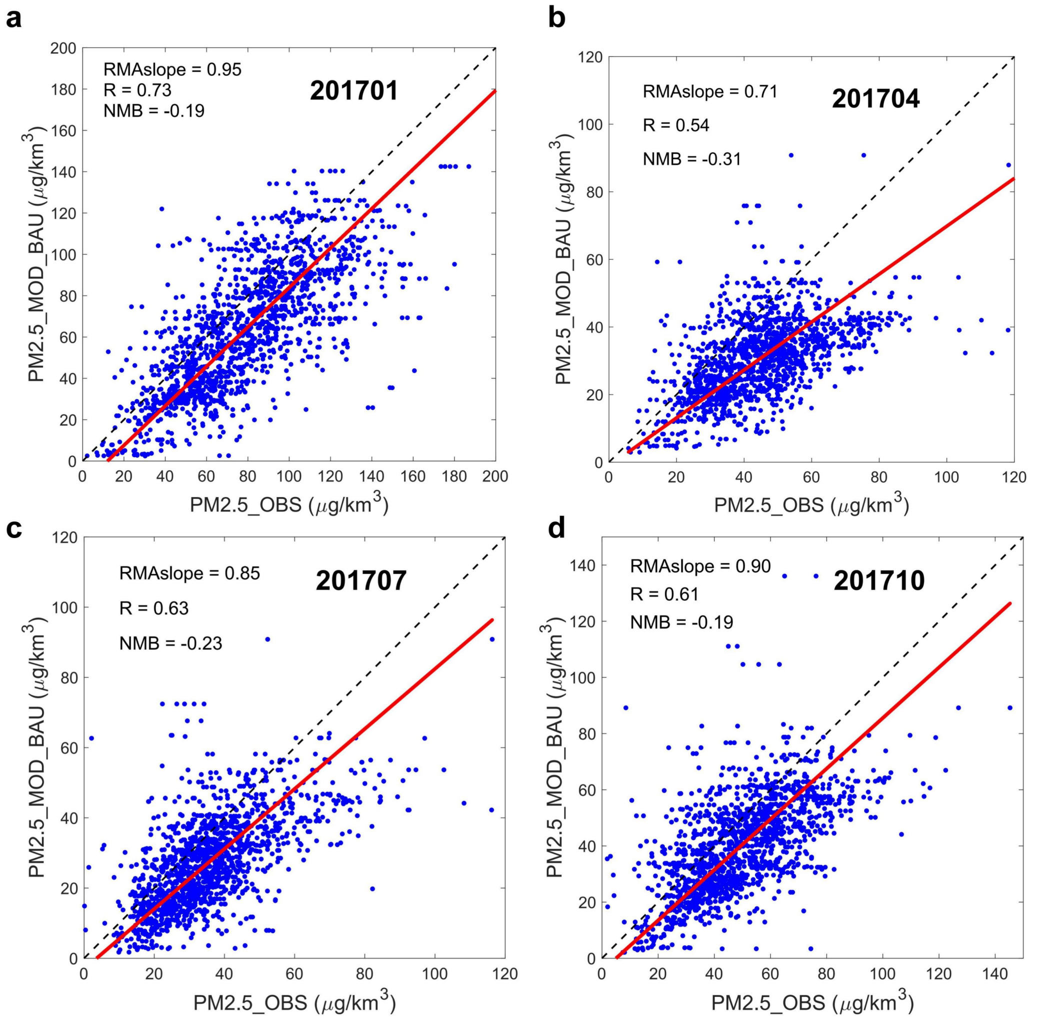

Fig. S10 | Comparison of observed and modeled monthly mean $PM_{2.5}$ concentrations. Correlation between modeled and actual observed $PM_{2.5}$ concentrations in January (**a**), April (**b**), July (**c**) and October (**d**) of 2017. The horizontal coordinates of the graph represent the actual observed values, while the vertical coordinates represent the modeled values by the WRF-Chem model.

Supplementary References:

1. Wang, S. et al. Urbanization can benefit agricultural production with large-scale farming in China. *Nat. Food* **2**, 183-191 (2021).
2. Duan, J. et al. Consolidation of agricultural land can contribute to agricultural sustainability in China. *Nat. Food* **2**, 1014-1022 (2021).
3. Zhu, Z. et al. Integrated livestock sector nitrogen pollution abatement measures could generate net benefits for human and ecosystem health in China. *Nat. Food* **3**, 161-168 (2022).
4. China Ministry of Agriculture and Rural Affairs. Technical Guide for Measuring the Land Carrying Capacity of Livestock and Poultry Manure (2018).
5. Gu, B., Ju, X., Chang, J., Ge, Y. & Vitousek, P.M. Integrated reactive nitrogen

budgets and future trends in China. *Proc. Natl Acad. Sci. USA* **112**, 8792-8797 (2015).
6. Gu, B. et al. Toward a Generic Analytical Framework for Sustainable Nitrogen Management: Application for China. *Environ. Sci. Technol.* **53**, 1109-1118 (2019).
7. Wang, C. et al. An empirical model to estimate ammonia emission from cropland fertilization in China. *Environ. Pollut.* **288**, 117982 (2021).
8. Wang, C. et al. Ammonia Emissions from Croplands Decrease with Farm Size in China. *Environ. Sci. Technol.* **56**, 9915-9923 (2022).
9. Zhou, M. et al. Environmental benefits and household costs of clean heating options in northern China. *Nature Sustainability* **5**, 329-338 (2022).
10. Xia, Y. et al. A new framework to model the distributed transfer and retention of nutrients by incorporating topology structure of small water bodies. *Water Res.* **238**, 119991 (2023).
11. Cheng, F.Y. & Basu, N.B. Biogeochemical hotspots: Role of small water bodies in landscape nutrient processing. *Water Resour. Res.* **53**, 5038-5056 (2017).
12. Xia, Y. & Yan, X. How Variations in Constructed Wetlands Geography Affect Nutrient Discharge. *Journal of Geophysical Research: Biogeosciences* **125** (2020).
13. Alexander, R.B., Smith, R.A. & Schwarz, G.E. Effect of stream channel size on the delivery of nitrogen to the Gulf of Mexico. *Nature* **403**, 758-761 (2000).
14. Zhang, X. et al. Societal benefits of halving agricultural ammonia emissions in China far exceed the abatement costs. *Nat. Commun.* **11**, 4357 (2020).
15. Lesiv, M. et al. Estimating the global distribution of field size using crowdsourcing. *Glob. Change Biol.* **25**, 174-186 (2019).
